# Human Parechovirus 3 and Neonatal Infections

**DOI:** 10.3201/eid1101.040606

**Published:** 2005-01

**Authors:** Guy Boivin, Yacine Abed, François D. Boucher

**Affiliations:** * Centre Hospitalier Universitaire de Québec and Laval University, Québec City, Québec, Canada

**Keywords:** Parechovirus-3, enterovirus, neonatal sepsis, hospitalization, PCR, research

## Abstract

A third serotype of human parechovirus (HPeV) has been recently isolated from stool specimens of a young Japanese child with transient paralysis. We report 3 additional cases of neonatal sepsis caused by HPeV-3 in the fall of 2001 in Canadian infants 7–27 days old. All children were hospitalized with high fever, erythematous rash, and tachypnea for a median of 5 days. The viruses isolated from nasopharyngeal aspirates grew slowly on tertiary monkey kidney cells and were successfully passaged on Vero cells. The predicted amino acid identity of the VP0-VP3-VP1 region of the three viruses was 74.6%–74.8%, 73.4%–73.6%, and 97.0%–97.1% when compared to HPeV-1, -2, and –3 prototype strains, respectively. Although different, our isolates were closely related; amino acid identity was 99.6%–100% for the last 3 proteins.

## Abstract 2

Three case reports expand the clinical spectrum of HPeV-3 infections and highlight the need to characterize this new pathogen.

Viruses belonging to the *Picornaviridae* family have been recently reclassified into 9 genera based on acid lability, serum neutralization, and sequence homologies (*1*). The *Parechovirus* genus is 1 of 5 picornavirus genera, along with enteroviruses, hepatoviruses, rhinoviruses, and kobuviruses, known to infect humans. Human parechoviruses (HPeV) 1 and 2, previously known as echoviruses 22 and 23, were first isolated in 1956 (*2*). These viruses, in particular, HPeV-1, have been associated with gastrointestinal and respiratory tract infections, as well as occasional cases of encephalitis and flaccid paralysis (*3**–**7*). A new serotype of HPeV (HPeV-3) was described in 2004; the strain was isolated from a stool specimen of a 1-year-old Japanese girl with transient paralysis (*8*). Preliminary HPeV-3 seroepidemiologic studies from Japan indicated a seroprevalence of 85% in children entering elementary school (*8*). However, clinical information related to this viral infection is limited, and the virus has not been reported outside Japan. We report 3 cases of neonatal infection caused by HPeV-3 in Canada.

## Case Reports

### Case 1

A 27-day-old boy, born at term, was hospitalized on September 5, 2001, for fever of unknown origin. The fever appeared the day before without respiratory or gastrointestinal symptoms. His father had had sinusitis, and his 2-year-old brother had had an upper respiratory tract infection in the preceding few days.

On initial examination, the baby was alert, with a temperature of 39.4°C, a respiratory rate of 40 breaths per minute, heart rate of 143 beats per minute, and oxygen (O_2_) saturation at 98% (in room air). Blood pressure was not recorded. Results of physical examination were otherwise normal, with no lung rales, normal tympanic membranes, and no rash. Leukocyte count was 4.5 x 10^9^ cells/L (30% neutrophils, 0% band forms, 60% lymphocytes), hemoglobin level was 123 g/L, and platelet count was 166 x 10^9^ cells/L. C-reactive protein was undetectable. Urinalysis results were normal. A lumbar puncture was performed, and analysis of the cerebrospinal fluid showed erythrocytes with no leukocytes, a glucose level of 2.8 mmol/L (normal 2.2–4.4 mmol/L), and a protein level of 0.44 g/L (normal 0.15–0.45 g/L). Chest radiograph results were normal. The patient was admitted with a diagnosis of probable viremia, and intravenous antimicrobial drugs (ampicillin and gentamicin) were administered until the results of blood cultures were available.

The following day, the infant experienced an episode of O_2_ desaturation (90%) with increased respiratory rate (70/min) and temperature (39.7°C). A maculopapular rash was also noted on the trunk and extremities. Chest radiograph results remained normal. Because of the infant's tachypnea, a nasopharyngeal aspirate (NPA) was obtained for human respiratory syncytial virus (RSV) antigen testing (Test Pack, Abbott Laboratories, Abbott Park, IL) and direct immunofluorescence assays (Bartels, Carlsbad, CA) for adenoviruses and parainfluenza viruses 1–3. Results of all rapid antigenic tests were negative. NPA was inoculated into numerous cell lines, including Madin Darby kidney, LLC-MK2 (tertiary monkey kidney), Hep-2, human foreskin fibroblast, Vero, Mink lung, human lung adenocarcinoma (A-549), human rhabdomyosarcoma, transformed human kidney (293), and human colon adenocarcinoma (HT-29) cells. The patient's condition gradually improved over the next 3 days, fever disappeared, and O_2_ saturation returned to normal. Four days after admission, the patient was afebrile and eupneic. A pale macular rash was still present on the trunk. The leukocyte count was 12.5 x 10^9^ cells/L (8% neutrophils, 1% band forms, and 75% lymphocytes), with hemoglobin at 138 g/L and a low platelet count of 101 x 10^9^ cells/L. Blood and cerebrospinal fluid cultures remained negative, and the urine culture was interpreted as bacterial contamination. The patient was discharged after 5 days of hospitalization with a presumptive diagnosis of viremia and mild reactive thrombocytopenia. Intravenous antimicrobial drugs were switched to oral amoxicillin for 5 more days. The viral culture of NPA was positive for a nonhemadsorbing virus (CAN01-81235) that initially grew on LLC-MK2 cells after 18 days of incubation.

### Case 2

A 20-day-old girl, born at term, was hospitalized on September 27, 2001, for high fever and irritability. She was seen 2 days earlier with an acute upper respiratory tract infection with rhinorrhea and conjunctivitis, for which she was receiving topical erythromycin. The day before her hospitalization, her mother also noted a diffuse macular eruption. The patient had been in contact with her 3-year-old brother who had an upper respiratory tract infection a few days before.

On initial physical examination, the patient was conscious but irritable. Her temperature was 39.0°C, blood pressure was normal for age, respiratory rate was 48 breaths per minute, and heart rate was 190 beats per minute. No O_2_ desaturation and no rales at lung auscultation were noted. A pale erythematous rash was present on the trunk and the limbs. Otitis media was not observed. At admission, she had a leukocyte count of 5.6 x 10^9^ cells/L with 36% neutrophils, 10% band forms, and 33% lymphocytes. Hemoglobin level was 104 g/L, and platelet count was 296 x 10^9^ cells/L. The C-reactive protein level was normal at 4 mg/L. Urinalysis results were normal, and examination of the cerebrospinal fluid showed 2 x 10^6^ leukocytes/L, a glucose level of 3.2 mmol/L, and a protein level of 0.53 g/L. Chest radiograph results were normal, and the child was hospitalized with a diagnosis of viremia or bacteremia. Intravenous ampicillin and gentamicin were administered after blood and urine samples were obtained for cultures.

The next day, the patient had an episode of O_2_ desaturation (89% in room air), which led to her transfer to the intensive care unit for O_2_ administration and monitoring. Her temperature was 39.4°C, with a respiratory rate of 60 breaths per minute and a heart rate of 210 beats per minute. The rash had disappeared by that time. Results of chest radiograph remained normal. NPA was obtained for rapid antigenic tests and viral culture. Antigenic test results were negative. The infant responded well to O_2_ supplementation and was discharged from the intensive care unit after 24 hours. Fever disappeared 3 days after admission, and respiratory rate and O_2_ saturation gradually normalized. On day 3, the leukocyte count was 13.5 x 10^9^ cells/L, with 13% neutrophils, 1% band forms, and 79% lymphocytes. The hemoglobin level was 102 g/L, with a platelet count of 180 x 10^9^ cells/L. Antimicrobial drugs were discontinued after 4 days, since blood, urine, and cerebrospinal fluid cultures remained negative. The infant was discharged on day 5 of hospitalization with a diagnosis of viral infection of the upper respiratory tract accompanied by conjunctivitis. Viral culture of the stools collected the day after admission was negative. Viral culture of the NPA specimen was positive for a nonhemadsorbing virus (CAN01-81554) after 14 days of incubation on LLC-MK2 cells.

### Case 3

A 7-day-old girl, who was born at term without complications, was brought to the hospital on December 16, 2001, for a recent onset of fever, irritability, and loss of appetite. Medical history showed that her mother had an upper respiratory tract infection 3 days before with cough, fever, sore throat, and a faint rash on her arms. She had been treated with clarithromycin for sinusitis during the last month of her pregnancy. The patient had a 2.5-year-old brother who was in good health.

On initial medical evaluation, the infant was irritable and refused breastfeeding but was conscious. She had no history of respiratory or gastrointestinal infections. Her rectal temperature was 39.2°C, with a heart rate of 150 beats per minute, blood pressure of 82/39 mm Hg, and a respiratory rate of 62 breaths per minute. No bulging of the fontanel was observed, and the lungs were clear on auscultation. An erythematous rash involved the trunk and axillary area without petechiae. Initial laboratory tests showed a leukocyte count of 8.0 x 10^9^ cells/L, with 71% neutrophils, 5% band forms, and 18% lymphocytes. Hemoglobin level was 162 g/L, and platelet count was 199 x 10^9^ cells/L. C-reactive protein level was normal at 11 mg/L. Urinalysis results were normal, and cerebrospinal fluid contained 147 (144 erythrocytes and 3 leukocytes) x 10^6^ cells/L, with a glucose level of 3.1 mmol/L and protein level of 0.74 g/L. A chest radiograph did not show lung infiltrates. The child was hospitalized with an initial diagnosis of bacteremia, and treatment with intravenous ampicillin and gentamicin was instituted after blood and urine samples were collected for culture.

The next day, the erythrodermialike rash became more diffuse and confluent. Rectal temperature was at 38.5 C, and the infant was tachypneic but with good O_2_ saturation (97%) in room air. A physical examination showed bilateral acute otitis media, which was confirmed by an ear-nose-throat specialist. The peripheral leukocyte count increased to 11.8 x 10^9^ cells/L, with 69% neutrophils and 20% lymphocytes, whereas the platelet count had dropped to 145 x 10^9^ cells/L. Liver transaminases were normal, and the chest radiograph showed no infiltrates. Because of the infant's tachypnea, an NPA was obtained. All rapid antigenic test results were negative, and the NPA was cultured for virus. At this point, the regimen was changed to cefotaxime and gentamicin.

Over the next 4 days, the patient's condition progressively improved: fever decreased, breastfeeding improved, and respiratory rate normalized. In addition, the rash gradually faded, and areas of desquamation were noted. On her last day of hospitalization (5 days after admission), the infant was afebrile and eupneic. The leukocyte count was 13.5 x 10^9^ cells/L, with 20% neutrophils and 66% lymphocytes, and the platelet count had dropped to 127 x 10^9^ cells/L. Results of a streptozyme test were negative. All bacterial cultures of specimens collected at the time of admission (urine, blood, and cerebrospinal fluid) were negative. The patient was discharged from the hospital with diagnoses of bilateral acute otitis media, cutaneous eruption related to a viral infection, and reactive thrombocytopenia. On follow-up, 2 days after discharge, the infant was in good health. Viral cultures of urine and stools collected during the hospitalization were negative. However, viral culture from the NPA specimen was positive for a nonhemadsorbing virus (CAN01-82853) growing in LLC-MK2 cells after 16 days of incubation.

## Laboratory Findings

All 3 viruses initially grew in LLC-MK2 cells after 14–18 days. On passage, however, the viruses grew rapidly (in ≈4–5 days) in Vero cells, with cytopathic effects similar to those of enteroviruses. The viruses could not be neutralized by pools of antisera against human enteroviruses or by specific antisera for HPeV-1 and -2 (formerly echovirus-22 and -23). Immunofluorescence assays using antibodies against common respiratory pathogens (influenza A and B, parainfluenza viruses 1–3, RSV, adenovirus) and reverse transcription–polymerase chain reaction (RT-PCR) assays for human metapneumovirus (*9*) were all negative.

More than 2 years after these patients were treated, and after the initial description of HPeV-3 (*8*), 2 RT-PCR assays for HPeV-3 were performed with infected Vero cell culture supernatants and were positive. The first set of primers amplified an 810-bp fragment between the 5´ untranslated region (5´ UTR) and the VP0 gene, whereas the second set amplified a 2,030-bp fragment between the VP0 and 2A genes of HPeVs as reported (*8*). Amplified products were sequenced with an automated DNA sequencer (ABI 377A; Perkin-Elmer Applied Biosystems, Foster City, CA), and the nucleotide and deduced amino acid sequences were aligned along with representative strains from other HPeVs by using ClustalW.

**Figure 1:**
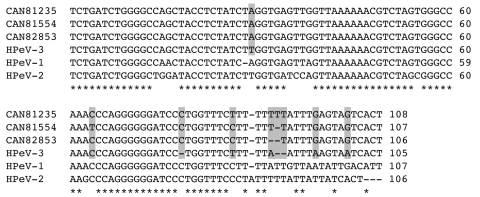
Comparison of the partial nucleotide sequences of human parechovirus (HPeV)-3 Canadian isolates no. 81235, 81554, and 82853 with reference sequences of HPeV-1, -2 and -3 (GenBank accession no. S45208, AF055846, and AB084913, respectively) for the 5´ untranslated region (5´ UTR; corresponding to nucleotides 595 to 699 of the HPeV-3 A308/99 strain, accession no. AB084913) (*8**,**10*). Asterisks denote identical nucleotides in all strains, whereas shaded nucleotides highlight differences between HPeV-3 isolates.

**Figure 2:**
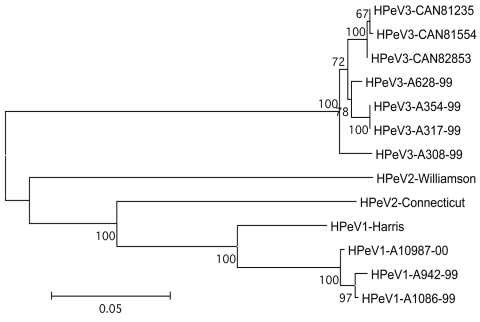
Phylogenetic tree showing the relationship between human parechovirus (HPeV)-3 Canadian isolates no. 81235, 81554, and 82853 and other HPeV-3 (A628-99, GenBank accession no. AB112484; A354-99, no. AB112483; A317-99, no. AB112482; A308-99, no. AB084913), HPeV-2 (Williamson, no. AJ005695; Connecticut, no. AF055846) and HPeV-1 (Harris, no. S45208; A10987-00, no. AB112487; A942-99, no. AB112486; A1086-99, no. AB112485) strains based on amino acid differences in capsid proteins (VP0-VP3-VP1 region). The tree was constructed by using the neighbor-joining method. Numbers represent the frequency of occurrence of nodes in 500 bootstrap replicas.

**Figure A1:**
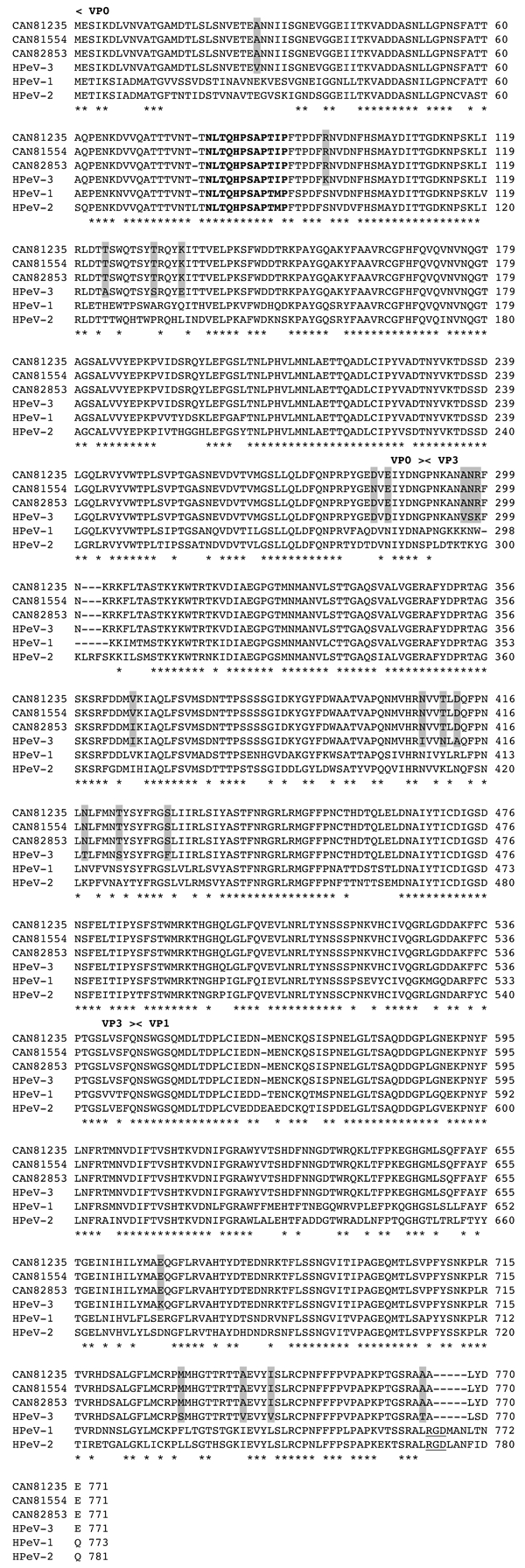
Comparison of the predicted amino acid sequences of human parechovirus (HPeV)-3 Canadian isolates no. 81235, 81554, and 82853 with reference sequences of HPeV-1, -2 and -3 (GenBank accession no. S45208, AF055846, and AB084913, respectively) for the VP0-VP3-VP1 proteins (*8**,**10*). Asterisks denote identical residues in all strains, whereas shaded amino acids highlight changes between HPeV-3 isolates. The RGD motif present only in HPeV-1 and -2 strains is underlined. The HPeV-1 immunodominant epitope described by Joki-Korpela et al. (*15*) is shown in bold.

When compared to prototype strains representative of HPeV-1 (GenBank accession no. S45208), HPeV-2 (no. AF055846), and HPeV-3 (no. AB084913), the nucleotide identity of the 3 Canadian strains for part of the 5´ UTR region was 88.0%–88.9% (HPeV-1), 80.6%–82.9% (HPeV-2), and 93.5%–94.3% (HPeV-3) (Figure 1). Nucleotide identity was 97.2%–98.1% between the 3 Canadian isolates for the same region. The nucleotide identity of the Canadian strains for the VP0-VP3-VP1 region (2,313 nucleotides, 771 amino acids) was 70.9%–71% (HPeV-1), 69.5%–69.5% (HPeV-2), and 96.0%–96.2% (HPeV-3). Similarly, the predicted amino acid identity for this region was 74.6%–74.8%, 73.4%–73.6%, and 97.0%–97.1% when compared to HPeV-1, -2 and -3 prototype strains, respectively (Figure A1). The Canadian strains were closely related, with nucleotide and amino acid identities of 99.2%–100% and 99.6%–100%, respectively, for these 3 capsid proteins. Contrasting with HPeV-1 and -2 strains, but similar to the HPeV-3 prototype, the RGD motif located near the C-terminus of VP1 was absent in all Canadian isolates (Figure A1). A phylogenetic tree confirming the relationship between Canadian HPeV strains and other HPeV-3 isolates is shown in Figure 2.

## Conclusion

We report the first 3 cases of HPeV-3 infection outside Japan and the first description of neonatal sepsis caused by this virus. The initial article on HPeV-3 infection by Ito et al. mentioned a case of transient paralysis with fever and diarrhea in a 1-year-old infant, as well as 3 other cases of gastroenteritis, exanthema, and respiratory illness, although no other clinical information was provided (*8*). In our study, viral infections occurred in the first month of life, and all patients had relatively similar clinical signs, including high fever, tachypnea, and erythematous rash. Acute otitis media and conjunctivitis were also noted in 1 patient each. All infants had a complete microbiologic work-up for possible bacteremia, including blood, urine and cerebrospinal fluid cultures, and all patients received at least 4 days of intravenous antimicrobial drugs for suspected bacterial sepsis. Neonatal infections caused by other HPeVs and many enteroviruses have been reported, including outbreaks of respiratory diseases on neonatal wards (*11*). Neonatal enteroviral infections can be severe, depending on the timing of maternal infection relative to time of delivery, and can progress to systemic disease, which may include hepatic necrosis, myocarditis, and necrotizing enterocolitis with or without neurologic involvement (*12*). Similar to some of our cases, a preceding maternal infection has been reported for 59% to 68% of neonates with enteroviral infections (*12*).

Most symptomatic HPeV-1 infections have been reported in young children, and most have been in children <1 year of age (*3**,**5*). For both HPeV-1 and the newly described HPeV-3, the proportion of seropositive children increases rapidly, reaching almost 100% by 5–6 years of age in Japan (*8**,**13*). HPeV-3 does not seem to be a recently emerging pathogen, as 87% of Japanese adults >40 years of age have preexisting antibodies (*8*). The reasons for the late description of this virus are unclear; as postulated for the newly described human metapneumovirus (*14*), a slow and restricted initial growth on a few cell lines (e.g., tertiary monkey kidney cells) might be one possibility, although additional epidemiologic and virologic studies are needed. In addition, as previously reported (*8*), HPeV-3 strains could not be neutralized with any antisera against human picornaviruses, including those directed against HPeV-1 and HPeV-2. The absence of the RGD motif near the C-terminus of the VP1 protein may be one of the epitopes involved in this event, since an antiserum specific for this region exhibited neutralizing activity against HPeV-1 (*15*). Also, the absence of the RGD motif in HPeV-3 is probably related to receptor usage since this region has been shown to interact with integrins (*16**,**17*). One of the HPeV-1 antigenic sites described by Joki-Korpela et al. (*15*), located near the N-terminal region of the VP0 protein (amino acid 79–90), was also conserved in our HPeV-3 strains as well as in reported HPeV-2 isolates (except for 1 residue), which suggests that it might represent a group antigen (Figure A1). All 3 Canadian strains isolated in the fall of 2001 were clearly related to the prototype HPeV-3 strain from Japan recovered in 1999 and reported in 2004 (97.0%–97.1% amino acid identity for the VP0-VP3-VP1 region), although point mutations could discriminate all our strains (Figure 1 and Figure A1).

In summary, we provide additional confirmation for a third serotype of HPeV that could not be neutralized with antisera against other known members of the *Parechovirus* genus. Our case reports expand the clinical spectrum of previously-reported HPeV-3 infections and highlight the need for additional research work to characterize clinical and epidemiologic features of this newly described pathogen. Of particular interest are the proportion of asymptomatic viral infections in childhood and the occurrence of reinfections in the adult population.
